# Electrolyte Disorders Associated with Piperacillin/Tazobactam: A Pharmacovigilance Study Using the FAERS Database

**DOI:** 10.3390/antibiotics12020240

**Published:** 2023-01-24

**Authors:** Heenam Seo, Eunyoung Kim

**Affiliations:** 1 Department of Pharmacy, Kangbuk Samsung Hospital, 29 Saemunan-ro, Jongno-gu, Seoul 03181, Republic of Korea; 2 Evidence-Based and Clinical Research Laboratory, Department of Health, Social and Clinical Pharmacy, College of Pharmacy, Chung-Ang University, 84 Heukseok-ro, Dongjak-gu, Seoul 06974, Republic of Korea; 3 The Graduate School for Regulatory Science, Chung-Ang University, 84 Heukseok-ro, Dongjak-gu, Seoul 06974, Republic of Korea; 4 The Graduate School for Pharmaceutical Industry Management, Chung-Ang University, 84 Heukseok-ro, Dongjak-gu, Seoul 06974, Republic of Korea

**Keywords:** piperacillin/tazobactam, hypokalemia, FAERS database, disproportionality analysis, data mining, pharmacovigilance, electrolyte disorder, penicillins

## Abstract

Electrolyte disorders (EDs) can disrupt normal bodily functions and lead to life-threatening complications. We evaluated whether piperacillin–tazobactam (TZP), a widely used antibiotic for moderate-to-severe infections, is associated with electrolyte imbalances via a disproportionality analysis of a self-reporting pharmacovigilance database. We searched The US Food and Drug Administration (FDA) Adverse Event Reporting System (FAERS) from 2004 to 2018 for EDs related to TZP and calculated three data-mining indices—the proportional reporting ratio (PRR), the reporting odds ratio (ROR), and the information component (IC)—compared to all other drugs. Signals were defined when one of the three criteria of the indices was met. For the signals detected in the initial analysis, further disproportionality analyses in relation to other penicillins were conducted using the same method. A total of 9829 reports related to TZP with 36,207 TZP–adverse event pairs were retrieved. Among 10 EDs, hypokalemia was detected as the only significant signal (PRR 2.61; ROR 2.61, 95% CI: 2.17–3.14; IC 95% lower CI: 1.11) compared to all other drugs. Compared with other penicillins, hypokalemia remained a significant signal for TZP using IC (95% lower CI: 0.26). In conclusion, TZP was significantly associated with hypokalemia.

## 1. Introduction

Electrolytes are essential for basic life functioning [1]. They play a crucial role in maintaining electrical neutrality in cells, generating and conducting action potentials in nerves and muscles including cardiac and respiratory muscles, and regulating osmolality and volume of body fluids and blood pressure [1,2]. Thus, electrolyte imbalances can cause a variety of medical problems, from mild abnormal body functions to life-threatening complications, depending on the involved electrolytes and severity.

Piperacillin/tazobactam (TZP) is a beta-lactam/beta-lactamase inhibitor combination [3]. It is the most widely prescribed antibiotic agent for moderate-to-severe infections owing to its broad-spectrum activity [3,4,5] and the option of carbapenem-sparing treatment [6]. TZP is considered a safe drug [4], but TZP-associated electrolyte disorders (EDs), including serious cases, have been widely reported [7,8,9,10,11,12,13,14,15,16,17]. Hypokalemia is the most frequently reported ED [7,8,9,10,11,12,13,14,15,16] with an inconsistent incidence, ranging from less than 1% to 24.8% [5,7,8,9]. Hypernatremia is another TZP-associated ED reported in the literature [14,17]. One study reported serious hypokalemia and hyponatremia following TZP therapy for peritonitis and peritoneal abscess [14]. Another study reported that hypernatremia was the most common side effect of TZP treatment in patients with sepsis [17]. In addition, Polderman et al. reported that piperacillin, a component of TZP, significantly decreased serum magnesium and potassium levels compared with other antibiotics in an intensive care unit [18]. 

Considering the broad use of TZP, it is important to evaluate TZP-associated key electrolyte disorders. We conducted a disproportionality analysis using a self-reporting pharmacovigilance database to detect signals of TZP-associated EDs in relation to all other drugs. For the EDs detected in the initial analysis, further analyses were performed to identify whether these EDs remained significantly associated with TZP compared to other penicillins (PCNs). Additionally, factors affecting EDs showing significant association in the initial analysis were explored. 

## 2. Results

### 2.1. General Characteristics 

A total of 11,933,093 spontaneous reports with 217,011,136 drug–adverse events (AEs) during the period from 2004 to 2018 were available on The US Food and Drug Administration (FDA) Adverse Event Reporting System (FAERS) database. Of these, 1,918,190 reports and 152,404,732 drug–AE pairs were excluded based on the exclusion criteria. The final drug–AEs amounted to 64,606,404 from 10,014,903 reports, of which 36,207 drug–AE pairs from 9829 reports were associated with TZP. 

The clinical characteristics of the TZP-associated reports are presented in Table 1. Patients were mostly adults (age ≥ 19 years). Males accounted for a larger proportion than females. Most of the reports were associated with serious outcomes (95.6%), of which hospitalization (51.1%) was the most frequent. Health professionals and France were the main reporting source and country, respectively.

### 2.2. Signal Detection

#### 2.2.1. Signal Detection of EDs Associated with TZP Compared to All Other Drugs

The numbers of reports of EDs and drug–AE pairs associated with TZP and all other drugs are listed in Table 2. In total, 304 TZP-associated EDs were reported. Hypokalemia was the most frequently reported ED associated with TZP. During the study period, 77,368 cases of hypokalemia were reported, of which 113 were related to TZP. In the disproportionality analyses relative to all other drugs, hypokalemia was the only ED detected for TZP using all three algorithms (proportional reporting ratio [PRR] 2.61; reporting odds ratio [ROR] 2.61, 95% confidence interval [CI]: 2.17–3.14; information component [IC] 95% lower confidence interval [LCI]: 1.11) (Table 2).

#### 2.2.2. Signal Detection of Hypokalemia Associated with TZP Compared to Other PCNs

Based on the results of our initial analysis, we performed a disproportionality analysis using hypokalemia for TZP compared to other PCNs. In total, 204,403 drug-AEs pairs from 55,933 reports for 12 PCNs, including TZP, were retrieved. As a result of the disproportionality analysis, hypokalemia was still detected as a signal for TZP; however, it was only detected using IC (IC 95% LCI: 0.26). Beta-lactamase-resistant PCNs, which are known as antistaphylococcal PCNs, showed strong signals compared to other PCN classes using all three algorithms as follows: flucloxacillin (PRR 2.48; ROR 2.49, 95% CI: 1.59–3.90; IC 95% LCI: 0.62); cloxacillin (PRR 3.59; ROR 3.61, 95% CI: 2.08–6.28; IC 95% LCI: 1.03); and nafcillin (PRR 9.05; ROR 9.20, 95% CI: 6.07–13.94; IC 95% LCI: 2.51) (Table 3). 

#### 2.2.3. Signal Detection of Hypokalemia Associated with PCNs Compared to All Other Drugs

In an additional disproportionality analysis of hypokalemia for 12 PCNs compared to all other drugs, TZP, which was already detected as a signal in the initial analysis, and other six PCNs—ampicillin (PRR 3.39; ROR 3.40, 95% CI: 1.21–2.32; IC 95% LCI: 1.20), piperacillin (PRR 2.83; ROR 2.83, 95% CI: 1.27–6.31; IC 95% LCI: 0.41), flucloxacillin (PRR 4.25; ROR 4.26, 95% CI: 2.15–6.62; IC 95% LCI: 1.45), cloxacillin (PRR 6.19; ROR 6.23, 95% CI: 3.61–10.74; IC 95% LCI: 1.85), oxacillin (PRR 3.37; ROR 3.38, 95% CI: 1.61–7.10; IC 95% LCI: 0.73), and nafcillin (PRR 15.15; ROR 15.41, 95% CI: 10.29–28.08; IC 95% LCI: 3.34)— showed strong signals using all three algorithms. Amoxicillin/clavulanic acid (IC 95% LCI: 0.18) and ampicillin/sulbactam (IC 95% LCI: 0.19) were only detected using IC (Table 4). Hypokalemia was identified as a signal in more PCNs when compared to all other drugs than when compared among PCNs.

### 2.3. Other Outcomes

Of 9829 reports related to TZP, only 8382 contained information regarding both sex and age. These reports were included in a logistic regression analysis to explore the risk factors for TZP-associated hypokalemia. Univariate logistic regression analysis identified female sex as a risk factor for hypokalemia and indicated that age did not affect hypokalemia development in TZP reports. Female sex remained a predictor of hypokalemia after adjusting for age (Table 5). As per the results of the Hosmer–Lemeshow test, the degree of fit was good (*p* = 0.847).

## 3. Discussion

Electrolytes play a vital role in maintaining basic homeostasis in the body [1]. EDs can interrupt normal bodily functions and may lead to life-threatening complications depending on their seriousness [1,2]. TZP is one of the most commonly used antibiotics for treating severely infected patients who are prone to electrolyte imbalances [3,4,5]. Therefore, it is necessary to determine whether TZP is associated with EDs. We conducted a disproportionality analysis using the FAERS database and found that hypokalemia was the only signal of 10 TZP-related EDs and remained a signal when compared to other PCNs. Hypokalemia is indicated as an adverse effect on the TZP label. Unexpected signals of EDs for TZP were not detected despite the presence of other ED-related reports. To our knowledge, this is the first study to evaluate TZP-related EDs using a pharmacovigilance database.

Hypokalemia is a common ED in clinical practice. Mild hypokalemia is generally asymptomatic; however, severe hypokalemia can result in life-threatening complications [19,20,21]. Cases of serious hypokalemia that caused arrhythmia or were refractory to potassium treatment and required discontinuation of TZP therapy have been widely reported [10,11,12,13,14,15,16]. Although TZP-associated hypokalemia is considered a rare adverse event, recently, a randomized clinical trial [7] and two observational studies [8,9] reported a remarkably high incidence of hypokalemia during TZP therapy (12.6%, 13.9%, and 24.8%, respectively). This study adds support to previous results by detecting the hypokalemia signal for TZP via a disproportionality analysis of a large real-world dataset.

Hypokalemia was identified as a signal in most PCNs compared to all the other drugs in our study. PCN-associated hypokalemia has been reported across penicillin classes [7,8,9,10,11,12,13,14,15,16,22,23,24,25,26,27,28,29,30,31]. Penicillins act as non-reabsorbable anions that generate a transmembrane potential gradient in the cortical collecting tubules and increase potassium secretion, leading to hypokalemia [21,23]. Studies [8,9,23,24,29] that assessed the incidence of hypokalemia associated with PCNs showed various related incidences. Studies that investigated TZP-associated hypokalemia reported incidences of 13.9% and 24.8%, respectively [8,9]. Other studies that assessed the incidence of hypokalemia with flucloxacillin reported a 23.7–42% incidence [23,24]. Another study that evaluated the safety of nafcillin and oxacillin, including the risk of hypokalemia, reported that the nafcillin group showed a higher incidence of hypokalemia (51%) than the oxacillin group (17%) [29]. The results of these studies indicated nafcillin is associated with a higher incidence of hypokalemia, followed by flucloxacillin, TZP, and/or oxacillin. Similarly, in this study, the signal strength of hypokalemia differed between each PCN and was almost consistent with the results of previous observational studies. Nafcillin showed the strongest signal, followed by flucloxacillin, TZP, and/or oxacillin, in comparison to all other drugs or other PCNs. This finding will provide useful information in selecting specific PCN among those with similar antibacterial spectra. For example, the antistaphylococcal PCNs nafcillin and oxacillin are recommended as the primary choice for severe and invasive methicillin-susceptible *Staphylococcus aureus* infection [32,33,34]. Considering the similar costs and the likely equal efficacy of these agents, clinicians may consider prescribing oxacillin over nafcillin for invasive methicillin-susceptible *Staphylococcus aureus* infection, especially in patients at a risk of developing hypokalemia [35]. 

Previous studies have reported several risk factors for developing hypokalemia. Female sex was a frequently mentioned risk factor for hypokalemia [8,36,37,38]. Our study confirmed these results. Most of the potassium in the body is found in muscle cells. In general, females have lower muscle mass than males. Thus, females might have low total exchangeable potassium levels, resulting in a higher risk for hypokalemia [36]. Older age has been reported as another risk factor owing to the accompanying low body mass, polypharmacy, and malnutrition [8,9,36,39]; however, this remains controversial, with one study reporting that younger age was a significant predictor of hypokalemia [40]. In this study, there was no significant difference among the age groups in the number of hypokalemic and non-hypokalemic reports. These results, however, should be interpreted with caution, because analysis with different variables was not performed, since the data provided were limited in scope.

This study had some limitations. First, as part of the pharmacovigilance data, under-reporting and reporting bias may exist. The reporting rate may vary depending on the AEs [41], but it is generally ~6% on average [42]. Specifically, non-severe or non-serious AEs may have been under-reported [43]. Most TZP-related reports included in this study were of serious cases. Second, there are duplicate reports in the FAERS database. Although we manually deleted all the duplicates that we detected, it is almost impossible to exclude all the duplicates because the same report may be submitted by different reporters with different identification numbers and at different times. Third, it has been suggested that AE-reporting increases over the first two years after regulatory approval of a drug, and then declines continuously (i.e., Weber effect) [44,45]. All the PCNs included in our study were launched long before 2004, and the use of some of them decreased due to the emergence of alternative agents. This may have affected the results of the disproportionality analysis for each PCN. Therefore, generalizing the findings should be done with caution, and well-structured comparative studies are warranted. Despite these limitations, this study is valuable, and its findings are noteworthy. Our study is the first to assess TZP-associated EDs using a pharmacovigilance database and includes the first analysis that evaluates the relative signals of hypokalemia among PCNs. These results will provide valuable information for selecting PCNs and improving their safe use. Moreover, given that this study used the FAERS database, which includes worldwide reports, and a long study period, from 2004 to 2018, we can guarantee the representativeness of the international population. 

## 4. Materials and Methods

### 4.1. Data Sources

The FAERS database, which contains information on AEs and medication error reports submitted to the FDA voluntarily by healthcare professionals and consumers, as well as mandatory reporting by manufacturers and distributors [46], was mined. We used reporting data files published by the FDA on a quarterly basis on its website from 2004 to 2018 [47].

This database system transitioned from the Legacy Adverse Event Reporting System (LAERS) to the current system on September 10, 2012. Some changes were introduced to the FAERS database structure and existing field contents. Both LAERS and FAERS data consist of seven data tables: patient demographics and administrative information (DEMO), drug information (DRUG), adverse events (REAC), patient outcomes (OUTC), report sources (RPSR), drug therapy start and end dates (THER), and indications for use/diagnosis (INDI) [48]. The main differences between the LAERS and FAERS data are the renaming of the key fields in the DEMO table: “isr” and “case” in LAERS to “primaryid” and “caseid” in FAERS, respectively. The “isr” and “primaryid” are the primary link fields (primary key) between the tables [48]. 

Drugs in the DRUG table are classified as primary suspect, secondary suspect, concomitant, or interacting [48]. AEs and indications in the REAC and INDI tables are coded according to the standardized terminology of a hierarchical system in the Medical Dictionary for Regulatory Activities (MedDRA) at the level of the preferred term (PT) [48,49]. Each report can include more than one drug and more than one AE, resulting in more than one drug–AE pair per report.

### 4.2. Targeted AE and Study Drugs

The study drug was TZP. All other drugs were used as comparators for signal detection of TZP-related EDs. The 10 most important and popular EDs were selected to assess TZP-associated EDs: hypokalemia, hyperkalemia, hyponatremia, hypernatremia, hypocalcemia, hypercalcemia, hypomagnesemia, hypermagnesemia, hypophosphatemia, and hyperphosphatemia. The MedDRA preferred terms used for the extraction of EDs were as follows: “hypokalaemia”, “blood potassium decreased”, “hyperkalaemia”, “blood potassium increased”, “hyponatraemia”, “blood sodium decreased”, “hypernatraemia”, “blood sodium increased”, “hypocalcaemia”, “blood calcium decreased”, “hypercalcaemia”, “blood calcium increased”, “hypomagnesaemia”, “blood magnesium decreased”, “hypermagnesaemia”, “blood magnesium increased”, “hypophosphataemia”, “blood phosphorus decreased”, “hyperphosphataemia”, and “blood phosphorus increased”.

The PCNs used as comparators are listed in Table 6. We selected PCNs that had been used in the United States during the study period and flucloxacillin, which was not available in the US but is commonly used in European Union countries.

### 4.3. Data Extraction

The quarterly LAERS and FAERS ASCII data files from 2004 to 2018 were downloaded from the FDA website. A case may have multiple versions, i.e., an initial case version and/or one or more follow-up case versions. The latest version of a case contains the most up-to-date information. Thus, the final version of each case was selected for the disproportionality analysis. In addition, specific reports regarded as erroneous on the FDA website were omitted [48]. Data reported in different units, such as age, were standardized. Cases that contained missing or inappropriate data in the identification key row, drug name, or reaction row were removed. Drugs reported as concomitant in the ‘role cod’ field of the DRUG table were excluded. 

### 4.4. Statistical Analyses

Cases were classified by a specific AE versus all other AEs and a specific drug exposure versus all other drug or other PCNs exposure. The specific AE was hypokalemia or one of the other EDs, and the specific drug was TZP or one of the other PCNs. Two-by-two contingency tables were generated for the disproportionality analysis. Disproportionality analysis is a statistical method used to detect AE signals. In this study, for each drug–AE pair, frequentist and Bayesian methods were used to calculate disproportionality using PRR, ROR, and the Bayesian confidence propagation neural network (BCPNN) of IC [50,51,52,53]. The PRR is the reporting rate of one specific event among all events for a given drug compared to those for all other drugs in the database [50]. For the PRR, a signal is detected if the frequency is ≥3 and PRR is ≥2 with an associated χ2 value ≥4 [51]. The ROR is the odds ratio of one specific AE versus all other events for a given drug compared to those for all other drugs in the database [52]. For the ROR, a signal is detected if the frequency is ≥3, ROR is ≥2 with an associated χ^2^ value ≥4, and the lower limit of the 95% two-sided confidence interval (CI) exceeds 1 [51,52]. The signal metric in BCPNN is IC, which can be additional information obtained on the probability of the event by specifying a drug, and a signal is considered when the lower 95% CI exceeds 0 [53]. In this study, AEs were defined as signals when at least one of three indices met the criteria indicated above. The characteristics of TZP-associated reports were expressed as the frequency and proportion of variables. To explore the risk factors for each ED detected as a signal, univariate and multivariate analyses were performed for age and sex using the enter method based on the reports that included information on both sex and age, and *p* < 0.05 was considered statistically significant. The Hosmer–Lemeshow test was used to examine the goodness-of-fit for logistic regression models, and *p* > 0.05 was considered statistically significant. Statistical analyses were conducted using PASW Statistics 18.0 (IBM, Armonk, NY, USA). This study was approved by the institutional review board of Kangbuk Samsung Medical Center (approval no. 2020-08-041). 

## 5. Conclusions

Hypokalemia was the only significant signal of TZP-associated EDs compared to all other drugs and was still significant when compared to other PCNs. 

## Figures and Tables

**Table 1:** Characteristics of TZP-associated reports.

Characteristics	Total (*n* = 9829)
Age, *n* (%)	
≤18 years old	647 (6.6)
19–64 years old	4389 (44.7)
≥65 years old	3513 (35.7)
Unknown	1280 (13.0)
Sex, *n* (%)	
Male	5140 (52.3)
Female	3668 (37.3)
Unknown	1021 (10.4)
Serious adverse event, *n* (%)	
Serious	9396 (95.6)
Hospitalization	4798 (51.1)
Fatal	1699 (18.1)
Life-threatening	566 (6.0)
Other	2333 (24.8)
Non-serious	433 (4.4)
Report source, *n* (%)	
Health professional	9204 (93.7)
Consumer	406 (4.1)
Other	70 (0.7)
Unknown	149 (1.5)
Reporter countries, *n* (%)	
France	2343 (23.8)
USA	1535 (15.6)
Japan	777 (7.9)
United Kingdom	776 (7.9)
Other	2461 (25)
Unknown	1937 (19.7)

TZP: piperacillin/tazobactam.

**Table 2:** Frequency of EDs and other AEs for TZP and all other drugs, and signal detection using disproportionality analysis relative to all other drugs.

EDs	TZP		All Other Drugs		PRR	ROR (95% CI)	IC 95% LCI	chi-Square with Yates Correction
	No. of EDs	No. of Other AEs	No. of EDs	No. of Other AEs				
Hypokalemia	113	36,094	77,255	64,492,942	2.61 *	2.61 (2.17–3.14) *	1.11 *	110.45
Hyperkalemia	48	36,159	227,018	64,343,179	0.38	0.38 (0.28–0.50)	−1.82	48.94
Hyponatremia	33	36,174	342,483	64,227,714	0.17	0.17 (0.12–0.24)	−3.04	131.60
Hypernatremia	35	36,172	44,850	64,525,347	1.39	1.39 (0.99–1.94)	−0.01	3.48
Hypocalcemia	22	36,185	155,848	64,414,349	0.25	0.25 (0.17–0.38)	−2.59	48.29
Hypercalcemia	7	36,200	99,053	64,471,144	0.13	0.13 (0.06–0.26)	−4.01	41.62
Hypomagnesemia	28	36,179	104,301	64,465,896	0.48	0.48 (0.33–0.69)	−1.60	15.39
Hypermagnesemia	1	36,206	11,599	64,558,598	0.15	0.15 (0.02–1.09)	−4.74	3.85
Hypophosphatemia	7	36,200	54,216	64,515,981	0.23	0.23 (0.11–0.48)	−3.14	17.26
Hyperphosphatemia	10	36,197	17,537	64,552,660	1.02	1.02 (0.55–1.89)	−0.85	0.01

EDs: electrolyte disorders; AEs: adverse events; TZP: piperacillin/tazobactam; PRR: proportional reporting ratio; ROR: reporting odds ratio; CI: confidence interval; IC: information component; LCI: lower confidence interval. Signals are indicated with *.

**Table 3:** Frequency of hypokalemia and other AEs associated with PCNs, and signal detection using disproportionality analysis relative to other PCNs.

Drugs	PCNs		Other PCNs		PRR	ROR (95% CI)	IC 95% LCI	chi-Square with Yates Correction
	No. of Hypokalemia	No. of Other AEs	No. of Hypokalemia	No. of Other AEs				
Amoxicillin\Clavulanic acid	120	73,848	311	130,124	0.68	0.68 (0.55–0.84)	−0.67	12.67
TZP	113	36,094	318	167,878	1.65	1.65 (1.33–2.05)	0.26 *	20.85
Ampicillin\Sulbactam	12	5034	419	198,938	1.13	1.13 (0.64–2.01)	−0.64	0.07
Amoxicillin	81	59,895	350	144,077	0.56	0.56 (0.44–0.71)	−0.99	22.67
Ampicillin	26	6381	405	197,591	1.98	1.99 (1.34–2.96)	0.37 *	11.01
Piperacillin	6	1767	425	202,205	1.61	1.62 (0.72–3.62)	−0.42	0.84
Flucloxacillin	20	3913	411	200,059	2.48 *	2.49 (1.59–3.90) *	0.62 *	15.48
Cloxacillin	13	1742	418	202,230	3.59 *	3.61 (2.08–6.28) *	1.03 *	21.15
Oxacillin	7	1727	424	202,245	1.93	1.93 (0.92–4.09)	−0.09	2.24
Nafcillin	24	1299	407	202,673	9.05 *	9.20 (6.07–13.94) *	2.51 *	155.08
Penicillin V	5	5860	426	198,112	0.40	0.40 (0.16–0.96)	−2.49	3.90
Penicillin G	4	6412	427	197,560	0.29	0.29 (0.11–0.77)	−3.06	6.23

PCNs: penicillins; AEs: adverse events; PRR: proportional reporting ratio; ROR: reporting odds ratio; CI: confidential interval; IC: information component; 95% LCI: lower limit of 95% confidential interval. Signals are indicated with *.

**Table 4:** Frequency of hypokalemia associated with PCNs and all other drugs, and signal detection using disproportionality analysis in relation to all other drugs.

Drugs	PCNs		All Other Drugs		PRR	ROR (95% CI)	IC 95% LCI	chi-Square with Yates Correction
	No. of Hypokalemia	No. of Other AEs	No. of Hypokalemia	No. of Other AEs				
Amoxicillin\Clavulanic acid	120	73,848	77,248	64,455,188	1.36	1.36 (1.13–1.62)	0.18 *	10.82
TZP	113	36,094	77,255	64,492,942	2.61 *	2.61 (2.17–3.14)*	1.11 *	110.45
Ampicillin\Sulbactam	12	5034	77,356	64,524,002	1.99	1.99 (1.13–3.50)	0.19 *	4.93
Amoxicillin	81	59,895	77,287	64,469,141	1.13	1.13 (0.91–1.40)	−0.15	1.05
Ampicillin	26	6381	77,342	64,522,655	3.39 *	3.40 (1.21–2.32) *	1.20 *	41.48
Piperacillin	6	1767	77,362	64,527,269	2.83 *	2.83 (1.27–6.31) *	0.41 *	5.38
Flucloxacillin	20	3913	77,348	64,525,123	4.25 *	4.26 (2.15–6.62) *	1.45 *	46.50
Cloxacillin	13	1742	77,355	64,527,294	6.19 *	6.23 (3.61–10.74) *	1.85 *	51.51
Oxacillin	7	1727	77,361	64,527,309	3.37 *	3.38 (1.61–7.10) *	0.73 *	9.44
Nafcillin	24	1299	77,344	64,527,737	15.15 *	15.41 (10.29–28.08) *	3.34 *	303.52
Penicillin V	5	5860	77,363	64,523,176	0.71	0.71 (0.30–1.71)	−1.67	0.33
Penicillin G	4	6412	77,364	64,522,624	0.52	0.52 (0.20–1.39)	−2.23	1.32

PCNs: penicillins; AEs: adverse events; TZP: piperacillin/tazobactam; PRR: proportional reporting ratio; ROR: reporting odds ratio; CI: confidence interval; IC: information component; LCI: lower confidence interval. Signals are indicated with *.

**Table 5:** Logistic regression analysis of risk factors for TZP-associated hypokalemia.

Variable	Hypokalemia	Non-Hypokalemia	Univariate Analysis		Multivariate Analysis	
			OR (95% CI)	*p* Value	OR (95% CI)	*p* Value
N (miss)	105 (8)	8277 (1439)				
Female sex, *n* (%)						
	60 (57.1)	3377 (41)	1.93 (1.31–2.87)	<0.001	1.94 (1.32–2.87)	<0.001
Age (year), *n* (%)						
≤18	9 (8.6)	632 (7.6)	Reference		Reference	
19–64	47 (44.8)	4272 (51.6)	0.77 (0.40–1.69)	0.481	0.79 (0.40–1.72)	0.511
≥65	49 (46.7)	3373 (40.8)	1.02 (0.52–2.23)	0.956	1.04 (0.53–2.28)	0.908

OR: odds ratio; CI: confidence interval.

**Table 6:** Penicillins used in the disproportionality analysis and their classification based on the ATC code.

Classification (ATC Code)	Penicillins
Combinations of penicillins, including beta-lactamase inhibitors (J01CR)	Piperacillin\Tazobactam
	Amoxicillin\Clavulanic acid
	Ampicillin\Sulbactam
Penicillins with extended spectrum (J01CA)	Ampicillin
	Amoxicillin
	Piperacillin
Beta-lactamase-resistant penicillins (J01CF)	Cloxacillin
	Flucloxacillin
	Nafcillin
	Oxacillin
Beta-lactamase-sensitive penicillins (J01CE)	Penicillin G
	Penicillin V

ATC code: Anatomical Therapeutic Chemical code.

## Data Availability

Data are available on the FAERS database.
